# The plasma levels of 12 cytokines and growth factors in patients with gastric cancer

**DOI:** 10.1097/MD.0000000000010413

**Published:** 2018-05-11

**Authors:** Zhengyun Zou, Lianjun Zhao, Shu Su, Qin Liu, Lixia Yu, Jia Wei, Yang Yang, Juan Du, Jie Shen, Xiaoping Qian, Xiangshan Fan, Wenxian Guan, Baorui Liu

**Affiliations:** a The Comprehensive Cancer Center of Drum-Tower Hospital Affiliated to Medical School of Nanjing University, Clinical Cancer Institute of Nanjing University; b The Pathology Department of Drum-Tower Hospital Affiliated to Medical School of Nanjing University; c The General Surgery Department of Drum-Tower Hospital Affiliated to Medical School of Nanjing University, Nanjing, China.

**Keywords:** cytokine, gastric cancer, growth factor, inflammation, plasma

## Abstract

To assess the association of plasma cytokines and growth factor levels with clinical characteristics and inflammatory indices in patients with gastric cancer.

Plasma samples derived from 99 gastric cancer patients were used for analysis. Levels of interferon (IFN)-γ, tumor growth factor (TGF)-β1, tumor necrosis factor-α (TNF-α), interleukin (IL)-1β, IL-2, IL-4, IL-6, IL-8, IL-10, IL-12p40, IL-12p70, and vascular endothelial growth factor (VEGF) were measured by Luminex suspension array technology. The association between cytokine/growth factor levels and demographic/clinical characteristics was assessed. Correlation between cytokines and growth factor levels was assessed by Pearson's correlation analysis.

Male patients had significant higher levels of plasma TNF-α, IL-12p70, IL-4, IL-10, and VEGF as compared with those in women (*P* < .05). Plasma levels of TNF-α in older patients with gastric cancer (≥60 years) were higher than those in young patients (*P* < .05). Elevated plasma levels of IL-8 and IL-10 were identified as risk factors for increased tumor size (diameter ≥5 cm). Higher plasma levels of TGF-β1 were associated with increased risk of vascular or nerve invasion and advanced tumor stage. The levels of systemic inflammatory markers, including white blood cell counts, neutrophil/lymphocyte proportion, neutrophil-to-lymphocyte ratio, platelet-to-lymphocyte ratio (PLR), C-reactive protein and modified Glasgow prognostic score (mGPS) were closely associated with a series of plasma cytokines. A prominent correlation was observed between the plasma IL-12p70 and IFN-γ levels (*r* = 0.729, *P* < .01).

Our findings suggest that plasma cytokines and growth factor levels may help predict the development and progression of gastric cancer. Our findings need to be validated by larger studies.

## 1 Introduction

According to cancer statistics for 2015,^[1]^ gastric cancer was the second leading cause of cancer related death in China, next only to lung cancer. Although the potential mechanisms of carcinogenesis and progression of gastric cancer have not yet been fully understood, results of recent studies suggest that inflammatory changes in the tumor microenvironment play a vital role in carcinogenesis.^[2]^ Moreover, in addition to surgical resection and chemotherapy, immunotherapy has emerged as a promising therapeutic strategy for clinical management of gastric cancer.^[3]^

In the tumor-associated inflammatory microenvironment, immune cells communicate with each other through cytokines, chemokines, and growth factors, which may in turn promote or suppress tumor progression.^[4]^ Different types of immune cells have been shown to respond to a diverse range of biochemical signals. For instance, T helper (Th) 1 lymphocytes were shown to be activated by interferon (IFN)-γ and interleukin (IL)-12, both of which were generated mainly by dendritic cells.^[5]^ After induction, these cells generate proinflammatory cytokines including tumor necrosis factor-α (TNF-α) and IFN-γ.^[2]^ Th2 cells could be induced by IL-4, subsequently leading to a secretion of IL-10 which was an immunosuppressive cytokine.^[2]^ Epplein et al^[6]^ reported that high level of IL-8 was associated with increased risk of gastric cancer. Nevertheless, the association of plasma cytokines and growth factors with clinical characteristics and inflammatory indices of gastric cancer remains unexplored.

In this study, we assessed the plasma levels of 12 cytokines and growth factors in patients with gastric cancer using Luminex suspension array technology. An analysis was made to investigate the association of plasma cytokine and growth factor levels with clinical characteristics and inflammatory indices of gastric cancer patients. Our findings may provide important evidence of the potential involvement of these cytokines and growth factors in oncogenesis and development of gastric cancer.

## 2 Materials and methods

### 2.1 Subjects

A total of 105 individuals with pathologically confirmed gastric cancer who were admitted to the department of general surgery in Nanjing Drum Tower Hospital (NDTH) between March 2014 and September 2014, were enrolled. None of the patients had received prior surgical treatment, chemotherapy, radiotherapy, or any other anti-cancer therapy. Informed consent was obtained from all participants. The study was approved by NDTH Institutional Ethics Review Board.

Three days prior to surgery, fasting peripheral blood samples (2 mL) were collected from each patient. After centrifugation at 1500 rpm for 15 minutes, 100 μL of the supernatant was collected into a tube and stored at –80 °C for further use.

### 2.2 Inclusion criteria and exclusion criteria

**Figure 1:**
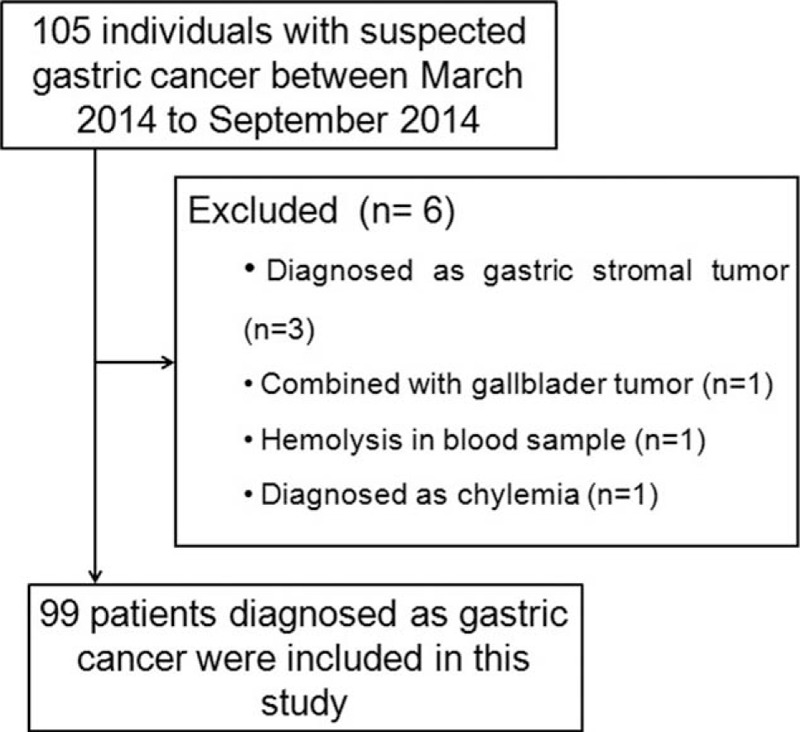
Schematic illustration of the selection criteria for patients with gastric cancer.

One hundred five patients diagnosed with pathologically confirmed gastric cancer were eligible for initial inclusion. Subjects who met any of the following criteria were excluded from the study: history of previous surgical treatment, chemotherapy, radiotherapy, or other anti-cancer therapies; patients with infection, chronic inflammation, or autoimmune disease at the time of blood collection; history of malignancy other than gastric cancer; presence of other severe disorders; evidence of hemolysis or other abnormalities in the collected blood samples. Six patients with gastric cancer were excluded, and a total of 99 patients were finally included in the analysis (Fig. 1).

### 2.3 Data collection

We collected the demographics of patients such as sex and age. The clinicopathological characteristics including tumor type, differentiation status, tumor diameter, vascular invasion, nerve invasion, tumor node metastasis (TNM) stage, T stage, N stage, were assessed by independent pathologists. The TNM stage was classified based on the 7th edition of the American Joint Committee on Cancer (AJCC) cancer staging system.^[7]^ To make further analysis in the present study, inflammatory indices in peripheral blood including the white blood cell counts, differential neutrophil counts, neutrophil-to-lymphocyte ratio (NLR), and platelet-to-lymphocyte ratio (PLR), C reactive protein (CRP), and modified Glasgow prognostic score (mGPS) were also tested or calculated.

### 2.4 Luminex suspension array technology

Plasma levels of IL-1β, IL-2, IL-4, IL-6, IL-8, IL-10, IL-12p40, IL-12p70, TNF-α, IFN-γ, TGF-β1, and vascular endothelial growth factor (VEGF) were measured by Luminex suspension array using a specific kit, according to manufacturer's instructions (Merck Millipore, Germany). In brief, 25 μL of plasma sample was mixed with 25 μL of Assay Buffer and 25 μL of beads and the mixture was loaded on 96-well plate and incubated at 4 °C in dark with shaking overnight. On the next day, the liquids in the 96-well plate were removed, and the plate was washed twice with 200 μL Wash Buffer. Subsequently, 25 μL of antibodies were added into each well and were incubated with 25 μL of PE-conjugated Streptavidin at room temperature for 30 minutes with shaking. The liquids were removed and the plate was washed twice with 200 μL Wash Buffer. Then, 100 μL of driving liquid was added into the plate and samples were analyzed with MagPlex instrument (Merck Millipore, Germany).

### 2.5 Statistical analysis

Statistical analysis was performed using SPSS 20.0 software. Data were expressed as median ± interquartile range. Non-parametric test was used to assess between-group differences. Correlation among different cytokines was evaluated by Pearson correlation analysis. Two-tailed *P* value <.05 was considered as statistically significant.

## 3 Results

### 3.1 Association of plasma cytokine and growth factor levels with demographic characteristics

In the whole population included in this study, plasma levels of TGF-β1 and VEGF were higher than those of the other cytokines. As shown in Table 1, plasma levels of TNF-α, IL-12p70, IL-4, IL-10, and VEGF in male patients (n = 75) were significantly higher than those in female patients (n = 24) (*P* < .05 for all). Patients aged ≥60 years had significantly higher plasma levels of TNF-α than those <60 years old (*P* < .05).

**Table 1:**
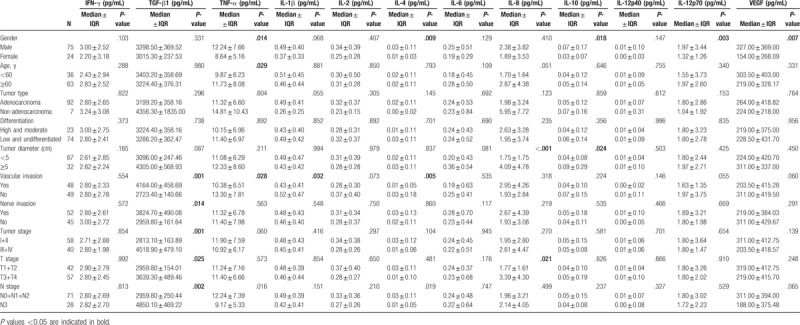
Profile of the plasma cytokines, growth factor levels with the demographic and clinical characteristics of patients with gastric cancer. Data are presented as median ± interquartile range (IQR).

### 3.2 Association of plasma cytokine and growth factor levels with clinicopathological parameters

We observed that plasma levels of cytokines and growth factors had no any association with tumor type and differentiation status (*P* > .05 for all) (Table 1). Patients with tumor diameter ≥5 cm had significantly higher plasma levels of IL-8 and IL-10 as compared with those with tumor diameter <5 cm (*P* < .05 for all). In addition, patients with vascular or nerve invasion showed higher plasma levels of TGF-β1, and lower plasma levels of TNF-α, IL-1β, and IL-4 as compared with those in patients with no invasion (*P* < .05 for all). Of note, plasma levels of TGF-β1 in patients with advanced T stage and N stage were higher than those in patients with early stage cancer; plasma IL-8 levels were also higher in patients with advanced T stage (*P* < .05).

### 3.3 Association of plasma cytokine and growth factor levels with inflammatory indices

We observed an increase in plasma levels of TGF-β1 and IL-6 with increase in the white blood cell count (**≥**4 × 10^9^ cells/L) (*P* < .05 for all) (Table 2). Likewise, a similar tendency was observed between the proportion of neutrophils and plasma levels of TNF-α, IL-6, IL-8, IL-10, IL-12p40, and IL-12p70, whereas the proportion of lymphocytes was inversely associated with plasma levels of IL-6, IL-10, and IL-12p70 (*P* < .05 for all). Plasma concentrations of IL-6, IL-8, IL-10 were found to be higher in patients with NLR value ≥3, and higher concentrations of IL-8 and IL-10 were also found in patients with PLR value ≥160 (*P* < .05 for all). Elevated CRP level (>8 mg/L) was found to be an indicator for increased plasma levels of IL-6, IL-8, and VEGF (*P* < .05 for all). Increased level of IL-4, IL-6, and IL-8 was also detected in the presence of higher plasma level of mGPS (*P* < .05).

**Table 2:**
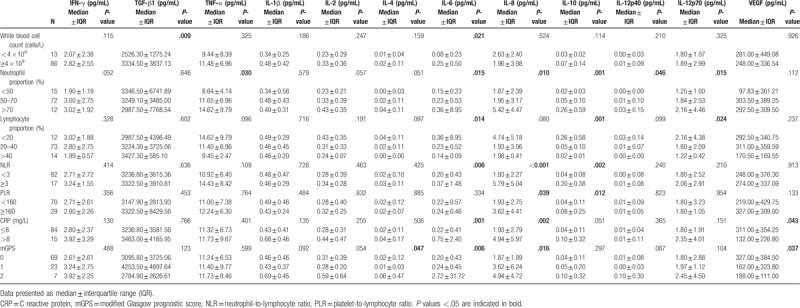
Profile of the plasma cytokines, growth factor levels with the inflammatory indices of gastric cancer.

### 3.4 Correlation analysis for plasma cytokines and growth factors

Pearson's correlation analysis revealed a significant correlation of plasma IL-2 level with IL-1β (*r* = 0.685, *P* < .01) and IL-12p70 (*r* = 0.603, *P* < .05) levels (Table 3). A more prominent positive correlation was observed between plasma IL-12p70 and IFN-γ levels (*r* = 0.729, *P* < .01).

**Table 3:**
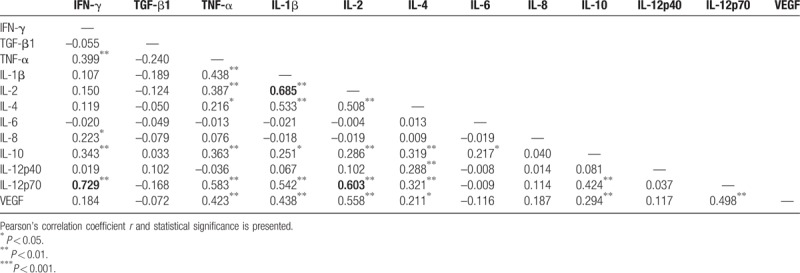
Correlation analysis of plasma levels of cytokines and growth factors in gastric cancer patients.

## 4 Discussion

In this study, we found that plasma levels of cytokines and growth factors had a close association with demographic and clinicopathological features including the inflammatory indices in patients with gastric cancer.

Plasma levels of TNF-α tended to vary by sex and age: male or old patients (≥60 years) had higher plasma TNF-α expression when compared with female and young patients, respectively. TNF-α is a well-characterized proinflammatory cytokine whose high expression might be a driver for carcinogenesis of gastric cancer.^[8]^ Increased TNF-α expression, possibly generated by the activated macrophages, was shown to be associated with inflammation and infection of gastric mucosa.^[9]^ Higher plasma levels of TNF-α in men as compared with that in women might be a potential mechanism underlying the observed 2-fold higher risk of gastric cancer among men.^[10]^ Moreover, the prevalence of abnormalities in gastric mucosa which were mostly induced by *Helicobacter pylori* (HP) infection, tended to increase with age, leading to an increased expression of IL-6, likely as an inflammatory response to HP infection.^[11]^ Therefore, men and older patients with gastric cancer might have increased circulating TNF-α levels induced by inflammatory response.

Among the various plasma cytokines, we observed the strongest correlation between plasma TGF-β1 and progression of gastric cancer. For instance, plasma TGF-β1 concentration was increased in patients with vascular invasion, nerve invasion, or advanced tumor stage. In line with our observations, TGF-β1 has been implicated in the development of epithelial-mesenchymal transition (EMT), tumor progression, and metastasis in the context of gastric cancer.^[12,13]^ Suda et al^[14]^ reported higher expressions of TGF-β1 in both plasma and tumor tissues with increase in tumor grade, which is consistent with the findings in the present study.

NLR, PLR, CRP, and mGPS values are well-accepted indices of systemic inflammatory response, all of which have been shown to have prognostic value for patients with gastric cancer.^[15,16]^ Gastric cancer patients with higher NLR level (≥3) were shown to have a poor prognosis^[17]^ and increased plasma levels of IL-6, IL-8, IL-10. Likewise, an unfavorable prognosis and higher plasma level of IL-8, IL-10 levels was also observed in the presence of higher PLR levels (≥160)^[15]^ in gastric cancer patients. The mGPS, assessed based on plasma level of CRP and albumin, was found to modulate the postoperative mortality risk in patients with gastric cancer.^[18]^ On the other hand, increased mGPS was also associated with advanced tumor stage of gastric cancer.^[18]^ In the present study, we found that higher mGPS was involved in increased plasma levels of IL-4, IL-6, IL-8. Increased levels of IL-4 and IL-8 may also be induced by *HP* infection, and therefore might play a role in the inflammatory response according to previous studies.^[19,20]^ In this study, 35 patients received HP examination. Among these, 12 were diagnosed as HP-positive, while others were negative for HP. None of the HP-positive patients received anti-bacterial or other treatment. Considering the close association between cytokine generation and HP infection in gastric cancer, future study will be conducted by recruiting a larger sample to address this issue. IL-6 is a key mediator of cancer-related inflammation^[21]^;high plasma levels of IL-6 were shown to predict a poor prognosis in patients with gastric cancer.^[4]^ Collecting these evidences, the important role in inflammatory response might be the potential mechanism for the observed association of cytokines with gastric cancer progression in the present study.

There are some limitations in this study. First, the retrospective study design is liable to introduce an element of bias. Second, although a series of plasma cytokines and growth factor levels were included in the analysis, some other important cytokines also likely to be involved in inflammatory responses may have been missed. Third, lifestyle-related variables, such as smoking, which may affect the levels of these cytokines, were not factored in the present study. Lastly, subjects included in this study were sourced from a single hospital and may not be representative of the whole population.

## 5 Conclusion

High plasma level of IL-6, IL-8, and TGF-β1 were predictors of gastric cancer progression. There was a close relationship between systemic inflammatory markers and plasma cytokines. Given the limitations of this study, large prospective studies are warranted to validate our findings.

## Author contributions

**Conceptualization:** Zhengyun Zou, Lianjun Zhao, Shu Su, Qin Liu, Jia Wei, Yang Yang, Juan Du, Jie Shen, Xiaoping Qian, Xiangshan Fan, Wenxian Guan, Baorui Liu.

**Data curation:** Zhengyun Zou, Lianjun Zhao, Shu Su, Qin Liu, Lixia Yu, Jia Wei, Yang Yang, Juan Du, Jie Shen, Xiaoping Qian, Xiangshan Fan, Wenxian Guan, Baorui Liu.

**Formal analysis:** Zhengyun Zou, Lianjun Zhao, Shu Su, Qin Liu, Lixia Yu, Yang Yang, Juan Du, Jie Shen, Xiaoping Qian, Xiangshan Fan, Wenxian Guan, Baorui Liu.

**Funding acquisition:** Zhengyun Zou, Lianjun Zhao, Shu Su, Qin Liu, Lixia Yu, Jia Wei, Yang Yang, Juan Du, Jie Shen, Xiaoping Qian, Wenxian Guan, Baorui Liu.

**Investigation:** Zhengyun Zou, Lianjun Zhao, Shu Su, Qin Liu, Lixia Yu, Jia Wei, Yang Yang, Jie Shen, Xiaoping Qian, Xiangshan Fan, Wenxian Guan, Baorui Liu.

**Methodology:** Zhengyun Zou, Lianjun Zhao, Shu Su, Qin Liu, Lixia Yu, Jia Wei, Yang Yang, Juan Du, Xiaoping Qian, Xiangshan Fan, Wenxian Guan, Baorui Liu.

**Project administration:** Zhengyun Zou, Lianjun Zhao, Shu Su, Qin Liu.

**Resources:** Zhengyun Zou, Lianjun Zhao, Shu Su, Qin Liu, Lixia Yu, Jia Wei, Yang Yang, Juan Du, Jie Shen, Xiaoping Qian, Xiangshan Fan, Wenxian Guan, Baorui Liu.

**Software:** Zhengyun Zou, Lianjun Zhao, Shu Su, Qin Liu, Lixia Yu, Jia Wei, Yang Yang, Juan Du, Jie Shen, Xiaoping Qian, Baorui Liu.

**Supervision:** Zhengyun Zou, Lianjun Zhao, Shu Su, Qin Liu, Xiangshan Fan.

**Validation:** Zhengyun Zou, Lianjun Zhao, Shu Su, Qin Liu, Lixia Yu, Jia Wei, Yang Yang, Juan Du, Jie Shen, Xiaoping Qian, Xiangshan Fan, Wenxian Guan, Baorui Liu.

**Visualization:** Zhengyun Zou, Lianjun Zhao, Shu Su, Qin Liu, Lixia Yu, Jia Wei, Yang Yang, Juan Du, Jie Shen, Xiaoping Qian, Xiangshan Fan, Wenxian Guan, Baorui Liu.

**Writing – original draft:** Zhengyun Zou, Lianjun Zhao, Shu Su, Qin Liu, Lixia Yu, Jia Wei, Yang Yang, Juan Du, Jie Shen, Xiaoping Qian, Xiangshan Fan, Wenxian Guan, Baorui Liu.

**Writing – review & editing:** Zhengyun Zou, Lianjun Zhao, Shu Su, Qin Liu, Lixia Yu, Jia Wei, Yang Yang, Juan Du, Jie Shen, Xiaoping Qian, Xiangshan Fan, Wenxian Guan, Baorui Liu.
